# Network-based analysis of genetic variants associated with hippocampal volume in Alzheimer’s disease: a study of ADNI cohorts

**DOI:** 10.1186/s13040-016-0082-8

**Published:** 2016-01-19

**Authors:** Ailin Song, Jingwen Yan, Sungeun Kim, Shannon Leigh Risacher, Aaron K. Wong, Andrew J. Saykin, Li Shen, Casey S. Greene, for the Alzheimer’s Disease Neuroimaging Initiative

**Affiliations:** Department of Genetics, Geisel School of Medicine at Dartmouth, Hanover, New Hampshire USA; Dartmouth-Hitchcock Norris Cotton Cancer Center, Lebanon, New Hampshire USA; Institute for Quantitative Biomedical Sciences, Dartmouth College, Hanover, New Hampshire USA; Center for Neuroimaging, Department of Radiology and Imaging Sciences, Indiana University School of Medicine, Indianapolis, Indiana USA; Center for Computational Biology and Bioinformatics, Indiana University School of Medicine, Indianapolis, Indiana USA; School of Informatics and Computing, Indiana University Indianapolis, Indianapolis, Indiana USA; Indiana Alzheimer Disease Center, Indiana University School of Medicine, Indianapolis, Indiana USA; Simons Center for Data Analysis, Simons Foundation, New York, NY USA; Department of Systems Pharmacology and Translational Therapeutics, Perelman School of Medicine, University of Pennsylvania, Philadelphia, Pennsylvnia USA

## Abstract

**Background:**

Alzheimer’s disease (AD) is a neurodegenerative disease that causes dementia. While molecular basis of AD is not fully understood, genetic factors are expected to participate in the development and progression of the disease. Our goal was to uncover novel genetic underpinnings of Alzheimer’s disease with a bioinformatics approach that accounts for tissue specificity.

**Findings:**

We performed genome-wide association studies (GWAS) for hippocampal volume in two Alzheimer’s Disease Neuroimaging Initiative (ADNI) cohorts. We used these GWAS in a subsequent tissue-specific network-wide association study (NetWAS), which applied nominally significant associations in the initial GWAS to identify disease relevant patterns in a functional network for the hippocampus. We compared prioritized gene lists from NetWAS and GWAS with literature curated AD-associated genes from the Online Mendelian Inheritance in Man (OMIM) database. In the ADNI-1 GWAS, where we also observed an enrichment of low *p*-values, NetWAS prioritized disease-gene associations in accordance with OMIM annotations. This was not observed in the ADNI-2 dataset. We provide source code to replicate these analyses as well as complete results under permissive licenses.

**Conclusions:**

We performed the first analysis of hippocampal volume using NetWAS, which uses machine learning algorithms applied to tissue-specific functional interaction network to prioritize GWAS results. Our findings support the idea that tissue-specific networks may provide helpful context for understanding the etiology of common human diseases and reveal challenges that network-based approaches encounter in some datasets. Our source code and intermediate results files can facilitate the development of methods to address these challenges.

**Electronic supplementary material:**

The online version of this article (doi:10.1186/s13040-016-0082-8) contains supplementary material, which is available to authorized users.

## Findings

Alzheimer’s disease (AD) is a neurodegenerative disease that affects memory and behavior. Around 70 % of the risk is expected to be attributable to genetics [1]. Including the well documented association with the gene *APOE*, previous studies have identified 22 susceptibility loci for late-onset Alzheimer’s disease (LOAD), of which 11 were recently reported by a large meta-analysis [2]. Most existing genetic analyses lack the affected tissue context, which is crucial to the precise actions of genes, though some eQTL analyses have begun to address this problem. For example, analysis of eQTLs in brain samples from control individuals and those with LOAD identified variants associated with changes in gene expression in the brain [3].

To identify genes that play a tissue-specific role in disease, we used the recently developed network-wide association study (NetWAS) method, which takes the tissue-specific functional roles of genes into account, to identify genes associated with differences in hippocampal volume [4]. We applied the NetWAS approach to genome-wide association study (GWAS) results from ADNI using hippocampal volume as an endophenotype. NetWAS identifies features in the hippocampus tissue-specific network that are associated with nominally significant GWAS *p*-values, and it uses these features to identify potentially novel AD gene associations. We evaluated the extent to which the NetWAS approach using a hippocampus network could identify AD-associated genes and compared results to the GWAS alone.

### Data

Data used in this study were obtained from the Alzheimer’s Disease Neuroimaging Initiative (ADNI) database (adni.loni.usc.edu). The ADNI was launched in 2003 by the National Institute on Aging (NIA), the National Institute of Biomedical Imaging and Bioengineering (NIBIB), the Food and Drug Administration (FDA), private pharmaceutical companies and non-profit organizations, as a $60 million, 5-year public-private partnership. The primary goal of ADNI has been to test whether serial magnetic resonance imaging (MRI), positron emission tomography (PET), other biological markers, and clinical and neuropsychological assessment can be combined to measure the progression of mild cognitive impairment (MCI) and early AD. Determination of sensitive and specific markers of very early AD progression is critical to aid researchers and clinicians to develop new treatments and monitor their effectiveness, and to reduce the time and cost of clinical trials.

The Principal Investigator of this initiative is Michael W. Weiner, MD, VA Medical Center and University of California - San Francisco. ADNI is the result of efforts of many co-investigators from a broad range of academic institutions and private corporations, and subjects have been recruited from over 50 sites across the U.S. and Canada. The initial goal of ADNI was to recruit 800 subjects but ADNI has been followed by ADNI-GO and ADNI-2. To date these three protocols have recruited over 1,500 adults, ages 55 to 90, to participate in the research, consisting of cognitively normal older individuals, people with early or late MCI (EMCI or LMCI), and people with early AD. The follow up duration of each group is specified in the protocols for ADNI-1, ADNI-2 and ADNI-GO. Subjects originally recruited for ADNI-1 and ADNI-GO had the option to be followed in ADNI-2. Thousands of longitudinal imaging scans [5, 6], performance on neuropsychological and clinical assessments [7] and biological samples [8] were collected at baseline and at follow-up visits for all or a subset of participants. Genome-wide genotyping data [9] are available on the full ADNI sample. For up-to-date information, see www.adni-info.org.

Genotype data of all participants from both ADNI-1 and ADNI-2 were downloaded, quality controlled, imputed to the Illumina 610Quad platform and combined, following the procedure described in [10]. Freesurfer version 5.1 was used to extract hippocampal volume and intracranial volume (ICV) measures from the 1.5 T baseline MRI scans of the ADNI-1 participants, and from the 3 T baseline MRI scans of the ADNI-2 participants, as previously described in [11]. Siblings and participants without hippocampal volume were excluded. As a result, we included 748 subjects and 817 subjects in the ADNI-1 and ADNI-2 analyses, respectively. GWAS analyses were performed by PLINK v1.07 using a linear regression model covaried for age, gender, education and ICV.

### The analytic workflow

**Fig. 1:**
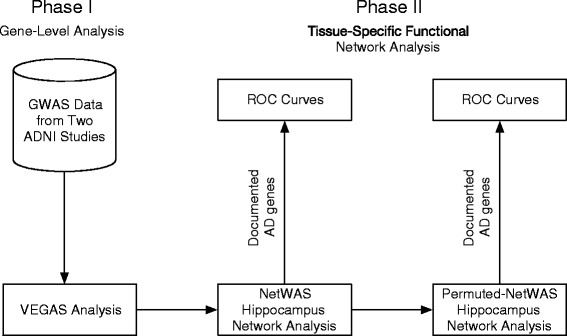
An overview of our analysis. In phase I, we mapped SNPs from the GWAS studies to genes and calculated gene-based *p*-values from SNP *p*-values. In phase II, we focused on using hippocampus network to reprioritize these genes. The efficacy of this approach was assessed with documented associations with AD. NetWAS results from different cohorts were integrated using an average-score approach and assessed for concordance with documented AD associations

Our analytical workflow was designed to identify genes associated with differences in hippocampal volume. Figure 1 provides an overview of our analytical strategy. To ensure reproducibility, we have used methods that are available from webservers and open source software packages to ensure accessibility of this analysis for other researchers. Because we performed a large number of NetWAS to evaluate the robustness of results, the actual analyses were performed on a computing cluster. We provide our source code and intermediate results under permissive licenses. The goal of phase I was to calculate gene-based association *p*-values based on SNP association *p*-values from the hippocampal volume GWAS. The goals of phase II were to reprioritize gene-based associations using hippocampus functional network and to assess the concordance of identified disease-gene associations with documented associations.

### Phase I: gene-level analysis

We applied the versatile gene-based association study (VEGAS) webserver to calculate gene-based *p*-values from individual SNP *p*-values, described in the data section above, for associations with hippocampal volume endpoint [12]. VEGAS assigns SNPs to individual genes using a window of 50 kb upstream and downstream from the gene sequence and generates a combined test-statistic that accounts for linkage disequilibrium (LD). The ADNI data we used were restricted to non-Hispanic Caucasian participants [10], so we used the HapMap CEU population for LD. This stage of analysis provided us with gene candidates associated with the hippocampal volume endpoint from the GWAS.

### Phase II: tissue-specific functional network analysis

The gene-based *p*-values calculated in phase I were used as input for a NetWAS analysis. NetWAS reprioritizes disease-gene associations using a functional network by constructing a support vector machine classifier using nominally significant genes (*p* < 0.01) as positive examples and non-associated genes as negative examples. The resulting classifier, which learned patterns in the biological network associated with nominal significance, was then applied back to the network to re-rank genes [4].

We performed NetWAS on the DISCOVERY supercomputer at Dartmouth. To evaluate the stability of predictions and estimate the variance of multiple NetWAS runs, we performed 1000 iterations of each analysis with randomly selected cross-validation intervals. To evaluate potential underlying bias in the method towards OMIM annotations, we also performed NetWAS on 1000 different GWAS for each cohort in which the associated genes had been permuted. Source code and intermediate and final results for these analyses are provided under permissive open source licenses [13] (code: BSD 3-clause; data: CC0).

The ultimate goal of this analysis was to use information about the tissue-specific functions and relationships of genes to identify disease-associated genes more effectively than GWAS. We mapped OMIM [14] annotations onto the Disease Ontology (DO) [15]. To assess the performance of this approach for this phenotype, we used the area under the receiver-operator characteristic curve (AUC) to compare NetWAS, permuted NetWAS and GWAS gene rankings. AUCs were calculated using annotations that were mapped to the DO term for Alzheimer’s disease (DOID:10652) as gold standard positives.

## Results and discussion

Using VEGAS, in phase I of the analysis we converted SNP-level association statistics from two different ADNI studies into two sets of gene-level association statistics for the hippocampal volume phenotype. These statistical associations were reprioritized in phase II using NetWAS analyses that leveraged a functional network for the hippocampus performed across 1000 independent sets of five-fold cross validation. To generate an aggregate list, we summed the ranks for each NetWAS. Tabular complete results for each of these analyses are available in the source code repository associated with this paper (ADNI-1 VEGAS: data/vegas_adni1.xls; ADNI-2 VEGAS: data/vegas_adni2.xls; NetWAS ADNI-1 and ADNI-2: combined-results.csv).

The underlying idea of NetWAS is that multiple truly associated genes may exhibit nominal, though not genome-wide, significance, which a machine learning method could use to extract phenotype-associated network features. Because NetWAS was developed to identify network commonalities among nominally associated genes, we generated quantile-quantile plots of observed VEGAS gene-wise *p*-values against a uniform distribution. ADNI-1 showed slight inflation, suggesting that this GWAS might represent a good NetWAS candidate (Additional file 1: Figure S1). ADNI-2 did not show evidence of inflation (Additional file 2: Figure S2).

**Fig. 2:**
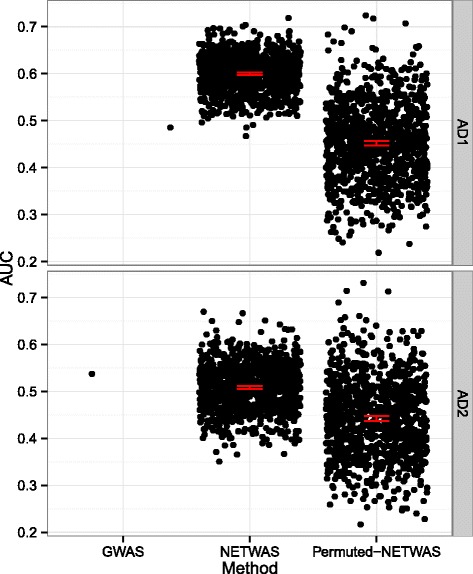
Network reprioritization of AD GWAS identifies AD-associated genes. GWAS ranked and NetWAS reprioritized genes are assessed for correspondence to genes known to be associated with Alzheimer’s disease. We compared ADNI-1 and ADNI-2 to DO-annotated AD genes using AUC. The AUC for NetWAS in ADNI-1 was significantly higher than that observed for the GWAS or the permuted NetWAS. The AUC for NetWAS in ADNI-2 was significantly higher than the permuted NetWAS. Error bars indicate 95 % confidence intervals via bootstrap

We compared the GWAS ranked and NetWAS reprioritized genes to genes with a documented association with Alzheimer’s disease in the OMIM database using the AUC (Fig. 2). Comparing the GWAS against the 95 % confidence intervals, we found that the NetWAS ranking more closely matched genes with a documented relationship to AD than genes identified by the initial VEGAS analysis in the ADNI-1 cohort. In the ADNI-2 cohort, this relationship was reversed but less pronounced. In both cases, the scores for the NetWAS performed using the GWAS associations were outside of and above the 95 % bootstrap confidence intervals for the mean of NetWAS with permuted gene-significance.

**Fig. 3:**
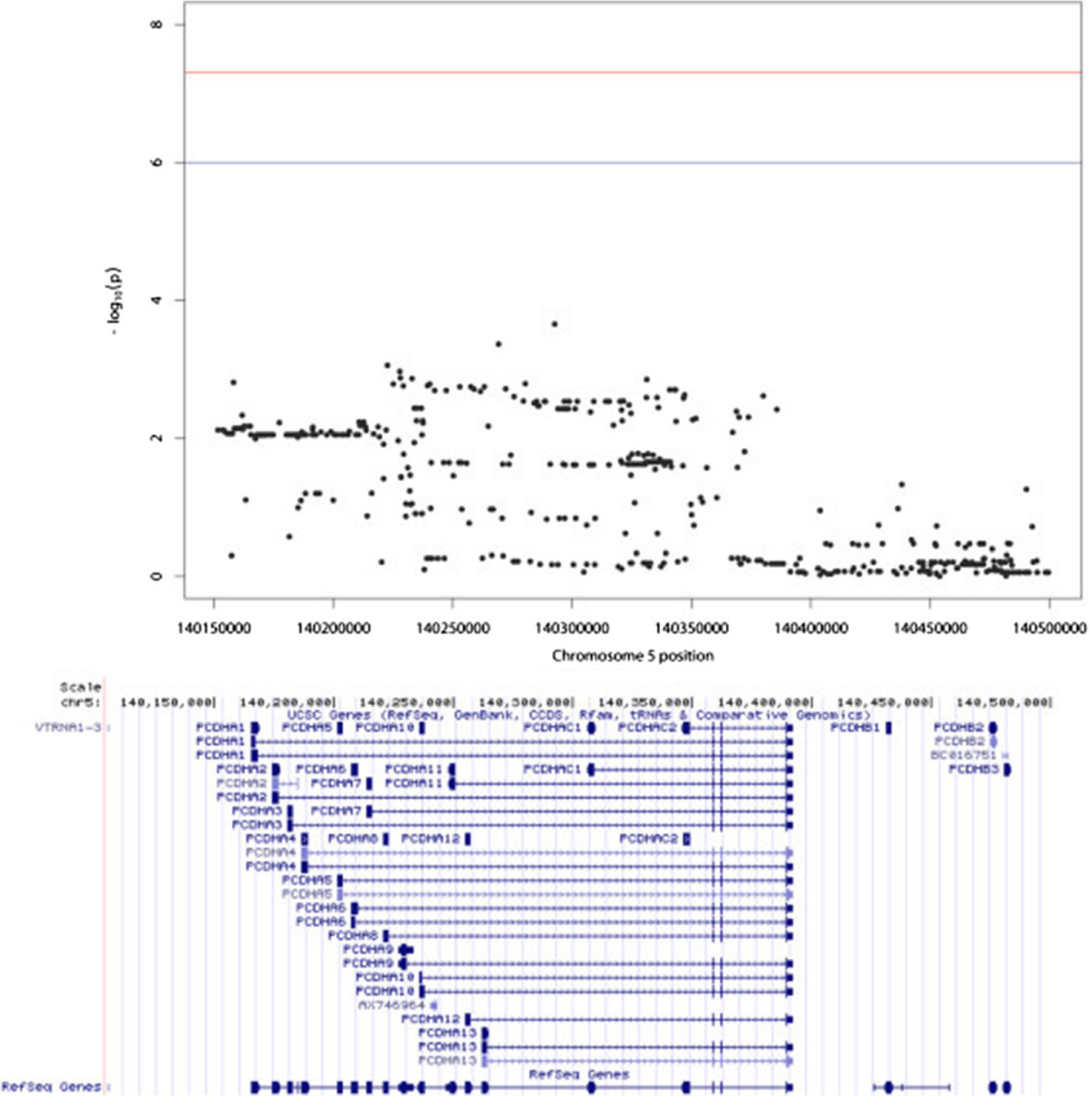
Significant SNP-wise *p*-values overlap with the entire *PCDHA* cluster. The Manhattan plot on the top shows *p*-values of the SNPs in the *PCDHA* region. The genome view at the bottom indicates where the genes are located in the genome. The overlap between significant SNPs and all *PCDHA* genes (*PCDHA1-13*) potentially explains the significant associations for all genes in this family so that they are largely identified by NetWAS

We noted that in the NetWAS results generated from ADNI-1 GWAS, the top 14 genes all belonged to the protocadherin alpha gene cluster (*PCDHA*). This unusual result had not previously been observed in a NetWAS analysis, so we investigated both network-based and association-based explanations. Examination of *p*-values from VEGAS revealed multiple significant *PCDHA* genes (*PCDHA1*-*13, PCDHAC1, PCDHAC2*). These associations could be driven by the same variants within the overall 50 kb window, as the *PCDHA* gene cluster includes multiple overlapping genes (Fig. 3). In the case that these genes are from the same family, as with *PCDHA* genes, they may also have similar network connectivity patterns, making it difficult for NetWAS to distinguish whether one or all members of the gene family should be most closely associated with the disease. The multiple associated genes by VEGAS also make it difficult for NetWAS to successfully distinguish involved members. These properties can, in certain circumstances, make it difficult to pinpoint the principally associated gene. This multiple-mapping problem has previously been reported to have impact on gene-set based analysis [16]. Our analysis demonstrates that users should also be cognizant of this for network-based approaches. Gene-based association tests that attempt to control for SNPs with potential associations with multiple genes may be required to address this challenge.

While a 50 kb window can lead to multi-gene mapping, it can also fail to capture long-range interactions between SNPs and genes. For example, SNPs 500 kb from the gene c-*MYC* were found to directly interact with the promoter of c-*MYC*, upregulating the expression of this gene [17]. Such relationships cannot be captured by existing gene-based association tests without creating additional multiple-mapping challenges. Gene-based association tests that account for potential long-range interactions through both haplotype structures and 3D chromosomal organization information would help to ameliorate this problem.

In summary, we have performed the first NetWAS for hippocampal volume, or any AD-related phenotype, in AD patients and controls using the ADNI-1 and ADNI-2 cohorts. In the course of this analysis, we identified the need for new gene-based association tests that aim to solve multiple-mapping challenges for SNP-gene relationships.

## Additional files

Additional file 1:
**Quantile-quantile plot showing the deviation of the distribution of p-values in the ADNI-1 dataset from the uniform expected under the null.** Some inflation was observed, consistent with gene-phenotype associations captured by the GWAS. (PDF 73 kb) Additional file 2:
**Quantile-quantile plot showing the deviation of the distribution of p-values in the ADNI-2 dataset from the uniform expected under the null.** We did not observe inflation of p-values for the ADNI-2 dataset. (PDF 77 kb)
