# Supplementary figures and images for: Network-based analysis of genetic variants associated with hippocampal volume in Alzheimer’s disease: a study of ADNI cohorts
Source: BioData Min. 2016 Jan 19;9:3. doi: 10.1186/s13040-016-0082-8 (PMC4717572; doi:10.1186/s13040-016-0082-8)

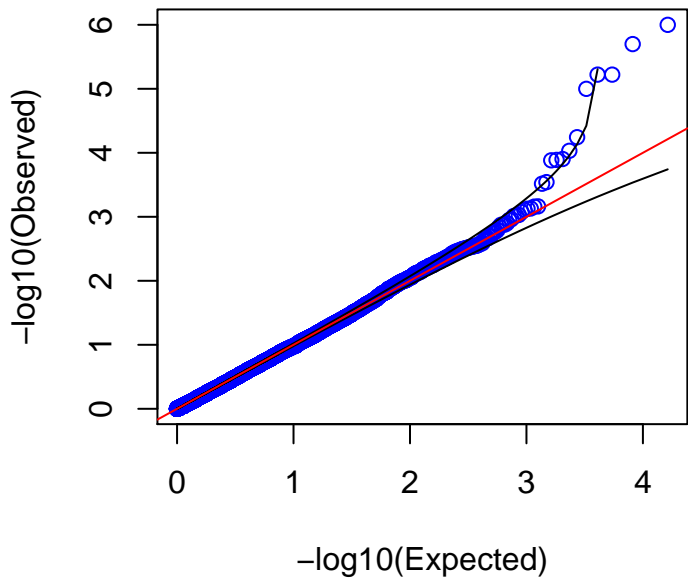

Supplement: Additional file 1: — Quantile-quantile plot showing the deviation of the distribution of p-values in the ADNI-1 dataset from the uniform expected under the null. Some inflation was observed, consistent with gene-phenotype associations captured by the GWAS. (PDF 73 kb) [file 13040_2016_82_MOESM1_ESM.pdf]

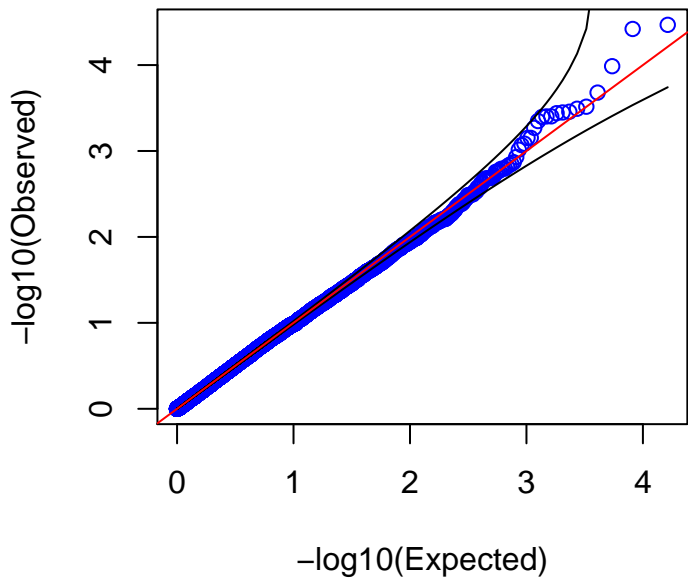

Supplement: Additional file 2: — Quantile-quantile plot showing the deviation of the distribution of p-values in the ADNI-2 dataset from the uniform expected under the null. We did not observe inflation of p-values for the ADNI-2 dataset. (PDF 77 kb) [file 13040_2016_82_MOESM2_ESM.pdf]
